# Human Platelet Lysate *versus* Fetal Calf Serum: These Supplements Do Not Select for Different Mesenchymal Stromal Cells

**DOI:** 10.1038/s41598-017-05207-1

**Published:** 2017-07-11

**Authors:** Eduardo Fernandez-Rebollo, Birgit Mentrup, Regina Ebert, Julia Franzen, Giulio Abagnale, Torsten Sieben, Alina Ostrowska, Per Hoffmann, Pierre-François Roux, Björn Rath, Michele Goodhardt, Jean-Marc Lemaitre, Oliver Bischof, Franz Jakob, Wolfgang Wagner

**Affiliations:** 1 Helmholtz-Institute for Biomedical Engineering, Stem Cell Biology and Cellular Engineering, RWTH Aachen University Medical School, Aachen, 52074 Germany; 2 Institute for Biomedical Technology – Cell Biology, RWTH Aachen University Medical School, Aachen, 52074 Germany; 3 Orthopedic Center for Musculoskeletal Research, Orthopedic Department, University of Würzburg, Würzburg, 97074 Germany; 4 Department of Genomics, Institute of Human Genetics, University of Bonn, Bonn, 53127 Germany; 5 Human Genomics Research Group, Department of Biomedicine, University of Basel, Basel, 4031 Switzerland; 6 Laboratory of Nuclear Organization and Oncogenesis, Department of Cell Biology and Infection, INSERM U.993, Institute Pasteur, 75015 Paris, France; 7 Department for Orthopedics, RWTH Aachen University Medical School, Aachen, 52074 Germany; 8 Institut Universitaire d’Hématologie, INSERM UMRS-1126, University Paris Diderot, 75010 Paris, France; 9 Institute of Regenerative Medicine and Biotherapies (IRMB), INSERM U1183, University of Montpellier, Montpellier, Cedex 05 34295 France

## Abstract

Culture medium of mesenchymal stromal cells (MSCs) is usually supplemented with either human platelet lysate (HPL) or fetal calf serum (FCS). Many studies have demonstrated that proliferation and cellular morphology are affected by these supplements – it is therefore important to determine if they favor outgrowth of different subpopulations and thereby impact on the heterogeneous composition of MSCs. We have isolated and expanded human bone marrow-derived MSCs in parallel with HPL or FCS and demonstrated that HPL significantly increases proliferation and leads to dramatic differences in cellular morphology. Remarkably, global DNA-methylation profiles did not reveal any significant differences. Even at the transcriptomic level, there were only moderate changes in pairwise comparison. Furthermore, the effects on proliferation, cytoskeletal organization, and focal adhesions were reversible by interchanging to opposite culture conditions. These results indicate that cultivation of MSCs with HPL or FCS has no systematic bias for specific cell types.

## Introduction

There is an unresolved controversy on whether mesenchymal stromal cells (MSCs) should rather be cultivated with the traditionally used fetal calf serum (FCS; alternatively termed fetal bovine serum [FBS]) or human platelet lysate (HPL)^1, 2^. So far, fully chemically defined culture conditions for MSCs remain elusive and this necessitates serum supplements - despite the obvious drawbacks with regard to standardization and quality control. There are some culture media on the market that claim to be serum free, but these do either not support the initial isolation of MSCs or they need to be used in combination with attachment substrates that contain human plasma. MSCs comprise a multipotent subset, capable of differentiation towards osteogenic, adipogenic, and chondrogenic lineage^3^. Due to their ease of isolation and potential immunoregulatory function MSCs represent the cell type that is currently used in most clinical trials. It is therefore important to better understand how the culture conditions impact on the cell preparations.

Fetal calf serum is usually considered as the gold standard for MSCs culture. However, there is high variation between FCS batches and it involves the risk of transmitting bovine infections or initiation of xenogeneic immune responses. More recently, HPL has been described as a viable alternative to FCS^4, 5^, enabling efficient propagation under animal serum-free conditions for clinical application – however with the drawback of possible transmission of human pathogens. HPL is enriched in growth factors and cytokines supporting the expansion of MSCs from bone marrow, umbilical cord blood, and adipose tissue. We have previously demonstrated that HPL of younger donors further accelerates proliferation as compared to HPL of older donors – but proliferation was anyway higher than in FCS^6^. In fact, the use of HPL facilitates generation of clinically relevant cell numbers already within two passages^7^.

Apart from the regulatory concerns and impact on proliferation it may be even more important to understand the biological sequel of these supplements on MSC preparations^8^. It has been demonstrated that culture conditions with either HPL or FCS give rise to MSCs with different morphological features^9, 10^. Given the heterogeneous composition of MSCs it may be anticipated that specific subpopulations are selected – or at least favored - by one or the other culture supplement. Furthermore, there are concerns that the high concentration of specific growth factors, such as platelet derived growth factors, may already drive MSCs towards specific lineages. Cellular differentiation is governed by epigenetic modifications, which impact on chromatin structure and regulate accessibility of specific genomic *loci* ^11^. The highly reproducible and quantitative differences in DNA-methylation (DNAm) patterns – which are apparently directly involved in cell type specification – provide an ideal basis for molecular characterization and definition of cell types^12^. In this study, we have therefore isolated and continuously cultured MSCs in parallel with HPL and FCS to directly compare their DNAm patterns and molecular characteristics.

## Results

### The supplements affect growth and morphology of MSCs

**Figure 1:**
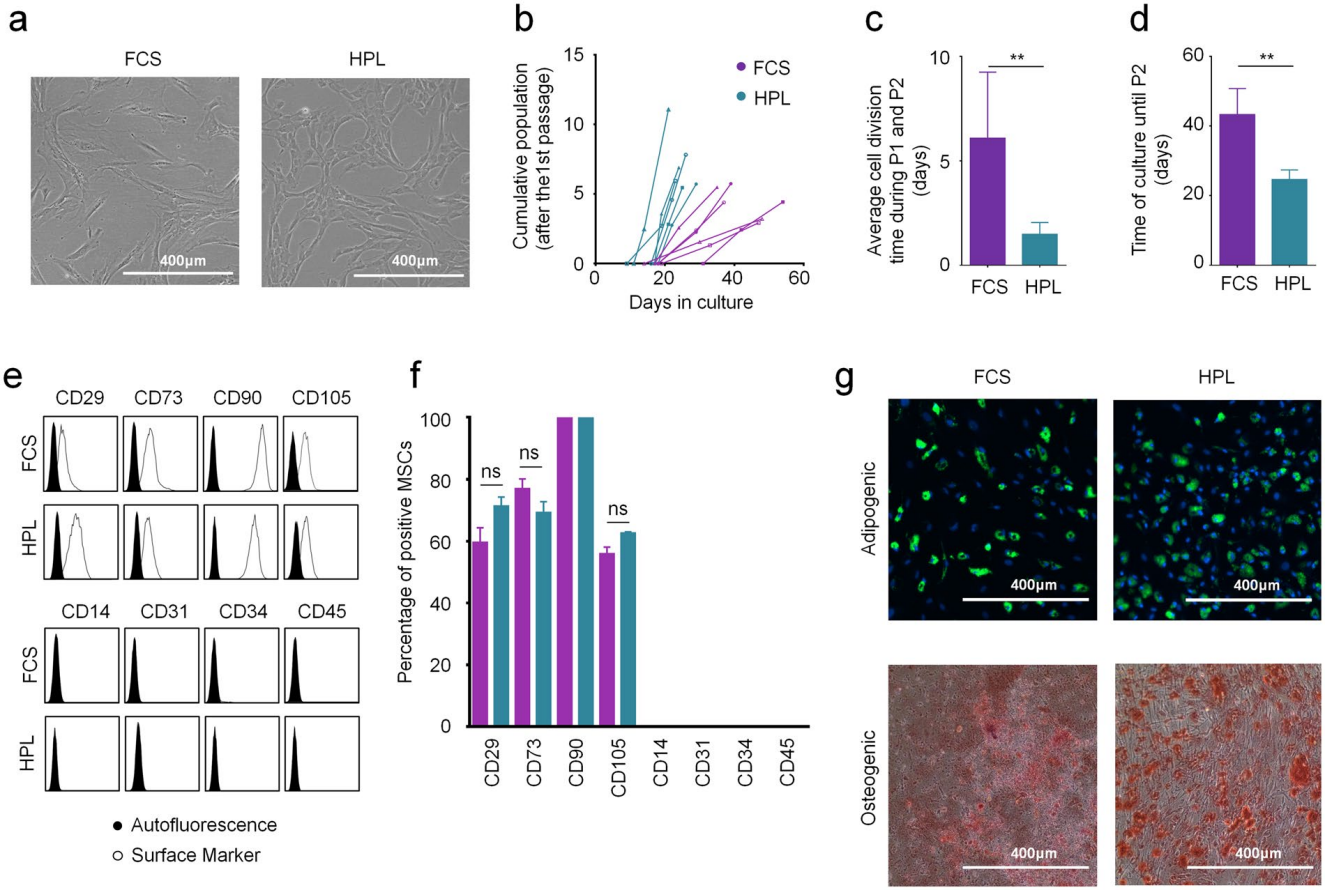
Growth and differentiation of MSCs in HPL and FCS. (**a**) Phase contrast images of MSCs (passage 2) that were in parallel cultivated in HPL and FCS. (**b**) Population doublings (PDs) within the first two passages were compared in HPL-MSCs and FCS-MSCs (n = 6). PDs in passage zero are not considered due to lack of initial cell numbers. (**c**) Average doubling time during passage 1 and 2 was shorter in HPL than FCS (**p < 0.01). (**d**) The time from initial isolation to passage two was shorter in HPL than in FCS (**p < 0.01). (**e**) Histograms depict the immunophenotype of MSCs isolated in FCS or HPL (autofluorescence in black). (**f**) The percentages of positive MSCs for the immunophenotypic markers did not differ in FCS or HPL. (**g**) HPL-MSCs and FCS-MSCs at passage 2 were in parallel induced with the same differentiation media for three weeks towards adipogenic or osteogenic lineage, and then stained for fat droplets or calcium precipitates (BODIPY/DAPI and alizarin red, respectively).

There are notoriously differences between batches of FCS and HPL. For our exploratory study we had to exemplarily select one well suited batch of FCS. Therefore, we have initially systematically compared 11 different FCS preparations from different companies to select a batch with reliable support of proliferation and *in vitro* differentiation for the subsequent experiments (Supplementary Figure S1). To reduce variation in HPL we always pooled platelet lysates of five apheresis products^2, 9^. MSCs were then isolated and expanded in parallel for two passages (n = 6) with either 10% FCS or 10% HPL. In HPL the MSCs revealed a more elongated spindle-shaped morphology, while FCS-MSCs were more flat (Fig. 1a), as described before^9^. Furthermore, cell growth was significantly accelerated in HPL as compared to FCS (Fig. 1b and c). With FCS the time to second passage was almost twice as long as in HPL (Fig. 1d). Viability of MSCs was very high in FCS and HPL and there was no apparent difference (Supplemental Figure S2). Pairwise comparison did not reveal significant immunophenotypic differences between HPL-MSCs and FCS-MSCs (Fig. 1e and f). Furthermore, the initial isolation with either HPL or FCS did not affect their differentiation potential towards osteogenic or adipogenic lineage, if these were induced in parallel with the same differentiation media (Fig. 1g). Chondrogenic differentiation was not performed since previous work demonstrated bone marrow-derived MSCs reveal similar chondrogenic differentiation potential^13, 14^. Nevertheless, the significant differences in proliferation rate and morphology may suggest that HPL-MSCs and FCS-MSCs constitute quite different cell preparations.

### DNA-methylation patterns do not differ in MSCs cultured in HPL or FCS

**Figure 2:**
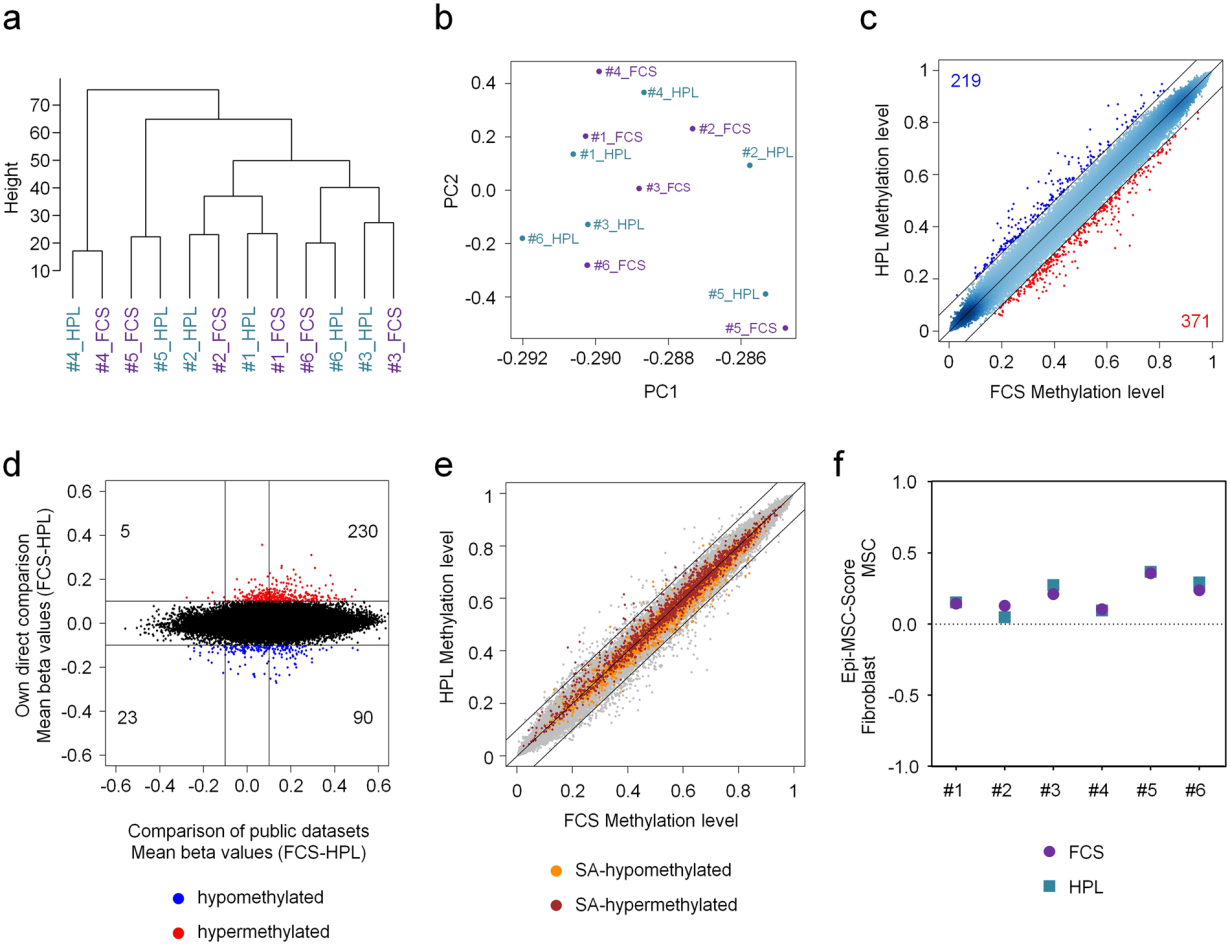
DNA-methylation patterns of MSCs are similar in HPL and FCS. (**a**) Hierarchical clustering of DNAm profiles (450 k BeadChips) discerned the six different MSC donors (n = 6). (**b**) Principal component (PC) analysis did not discern HPL-MSCs and FCS-MSCs. PC1 and PC2 are exemplarily depicted. (**c**) Scatter plot analysis of mean DNAm levels. CpGs that were at least 10% higher methylated in FCS or HPL are indicated in red and blue, respectively (none of the CpGs reached statistical significance). (**d**) Comparison of mean DNAm differences (FCS-MSCs/HPL-MSCs) in our study (y-axis) and publically available datasets (x-axis; 6 different studies, Supplementary Table S1). (**e**) This scatter plot depicts CpGs that either reveal senescence-associated hypermethylation (1,702 CpGs; red) or senescence-associated hypomethylation (2,116 CpGs; orange) during culture expansion of MSCs^15^. (**f**) Classification of cell preparations with the Epi-MSC-Score ^12^ confirmed that all cell preparations are MSCs.

Based on the above results, we reasoned that changes in proliferative capacity and cell morphology should be mirrored at the epigenome level. We therefore analyzed global DNAm profiles of MSCs (n = 6) after expansion for two passages in either FCS or HPL using Illumina's Infinium HumanMethylation450 k. Nonsupervised hierarchical clustering and principal component analysis (PCA) revealed donor-specific classification – but there was no systematic bias related to culture conditions (Fig. 2a and b). None of the CpGs revealed significant differences between MSCs that were cultured in HPL *versus* FCS if the analysis was corrected for multiple testing (limma-adjusted P-value of <0.05). These results indicate that there are no reproducible differences in DNAm patterns of HPL-MSCs and FCS-MSCs. To further determine, if there are moderate differences that might still be relevant, we used a less stringent cutoff of at least 10% difference in mean DNAm levels. With these thresholds 219 CpGs were hypomethylated, while 371 were hypermethylated in FCS-supplemented MSCs (Fig. 2c). These differences were then compared with 58 publicly available DNAm profiles of MSCs that were cultured with FCS or HPL (6 different studies, Supplementary Table S1). None of these studies has directly compared the two different culture conditions and therefore this cross-comparison might be influenced by other variables in experimental design or analysis. In fact, the DNAm differences of FCS-MSCs *versus* HPL-MSCs as observed in our study were hardly recapitulated in the comparison of publically available datasets (Fig. 2d).

The flattened and irregular shape of FCS-MSCs might reflect signs of cellular senescence – and this could be reflected by accelerated senescence-associated epigenetic modifications. *In vitro* culture of cells entails highly reproducible senescence-associated DNAm changes that can even be used to track passage numbers or population doublings^15, 16^. However, when we focused on senescence-associated CpG sites there was no clear acceleration with culture conditions (Fig. 2e). Supporting these results, staining of senescence-associated β-galactosidase (SA- β gal) and gene expression analysis of the senescence-associated genes *GLB1* (coding for SA- β gal), P53 (*TP53*), and P16 (*CDKN2A*) by qRT-PCR did not reveal differences in FCS-MSCs and HPL-MSCs (Supplemental Figure S3). Furthermore, we have recently described an epigenetic signature to classify MSCs *versus* fibroblasts^12^, and this Epi-MSC-Score further supported the notion that our cell preparations are MSCs (Fig. 2f). Overall, the DNAm profiles of MSCs were very similar despite continuous culture in either HPL or FCS – indicating that they are epigenetically alike.

### Gene expression profiling reveals only moderated differences between HPL-MSCs and FCS-MSCs

**Figure 3:**
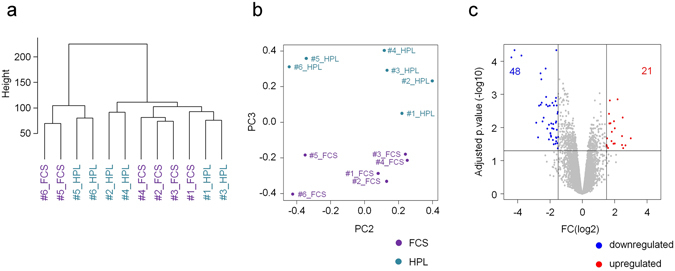
Differential gene expression of MSCs in HPL and FCS. (**a**) Hierarchical clustering of gene expression profiles (Affymetrix Human Transcriptome Array 2.0) does not separate cell preparations from different donors (n = 6), but rather by culture conditions. (**b**) Principal component analysis separates HPL-MSCs and FCS-MSCs, particularly in PC3. (**c**) Volcano plot of differential gene expression (fold change >1.5 and limma-adjusted P-value of <0.05). The number of differentially regulated transcripts is indicated (Supplementary Table S2).

Subsequently, we compared gene expression profiles of HPL-MSCs and FCS-MSCs (n = 6) with Affymetrix Human Transcriptome Arrays 2.0. In contrast to the DNAm profiles, hierarchical clustering of the gene expression profiles did not reveal donor association, but rather classification according to the supplements (Fig. 3a and b). However, when we apply a fold change >1.5 and limma-adjusted P-value of <0.05, only 69 transcripts were differentially expressed (48 upregulated and 21 downregulated; Fig. 3c; Supplementary Table S2). These genes were significantly enriched in gene ontology categories related to extracellular matrix (ECM; Supplementary Figure S4a). There was no clear association of differential gene expression and DNAm of corresponding promoter regions (Supplementary Figure S4b). The main deregulated genes were analyzed in MSCs that were isolated with two different FCS batches by qRT-PCR to rule out the possibility that the expression patterns are evoked by one specific FCS batch. Indeed the qRT-PCR results did not show statistical differences between the different FCS batches (Supplemental Figure S4c). In addition, we utilized gene expression profiles to further validate that senescence-associated genes are not differentially expressed in FCS-MSCs and HPL-MSCs (Supplementary Figure S5). With regard to the pronounced differences in growth and morphology of HPL-MSCs and FCS-MSCs it was unexpected that the differences in gene expression were rather moderate.

### Supplement-evoked differences in growth and morphology are reversible

**Figure 4:**
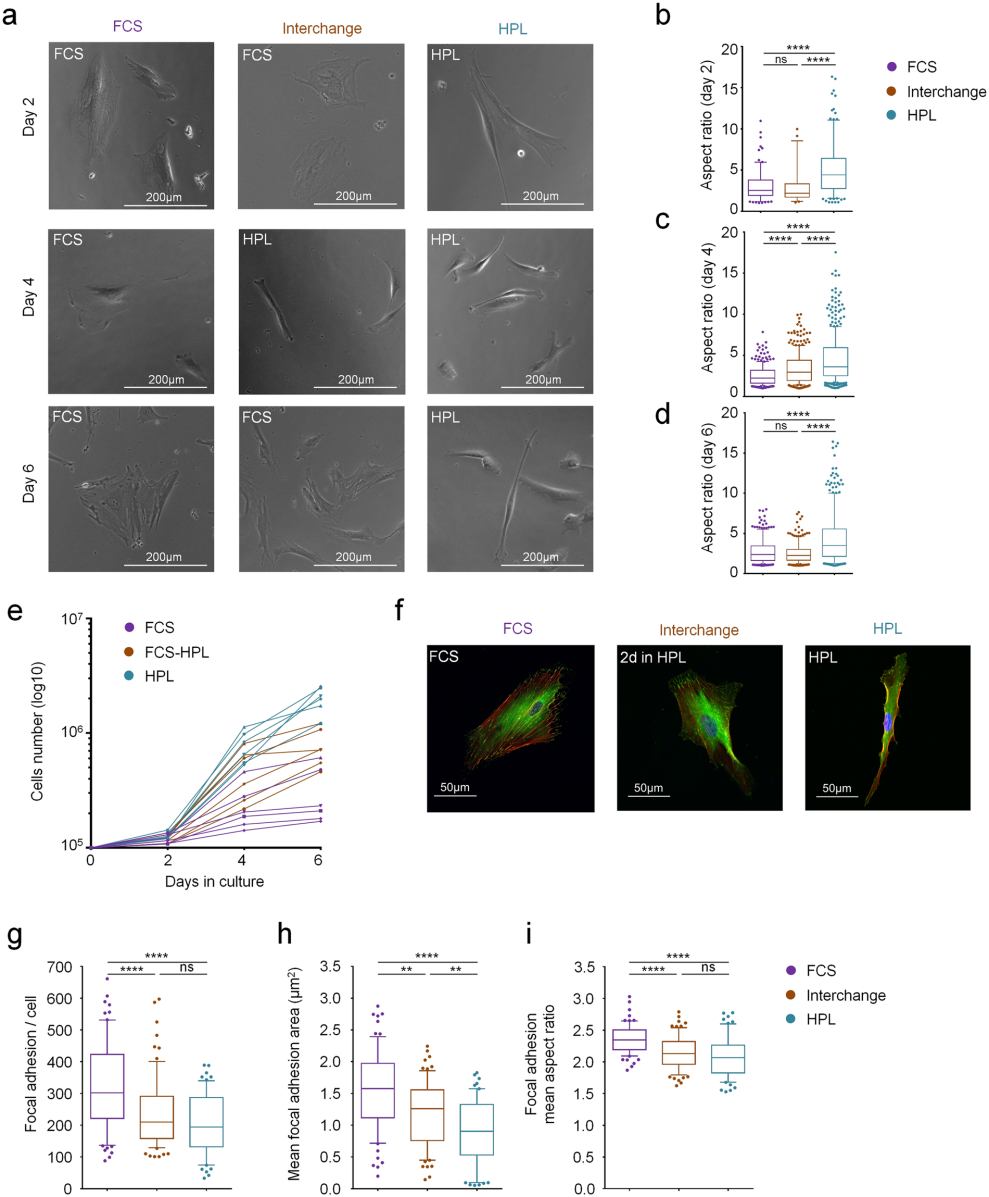
Growth of MSCs is reversible by interchanging the culture conditions. (**a**) To determine if effects of supplements are reversible, we have interchanged culture media from FCS (2 days) to HPL (2 days) and then back to FCS (2 days) (n = 4). Exemplary phase contrast images are provided. (**b**) Aspect ratios at day 2 demonstrate that HPL-MSCs are more elongated than FCS-MSCs (****p < 0.0001). (**c**) Aspect ratios at day 4 demonstrate that interchanging to HPL-medium for only two days already results in significant elongation of the cells (****p < 0.0001). (**d**) This change in morphology is again reversible by again changing for two days to FCS culture medium (day 6; ****p < 0.0001). (**e**) Proliferation can be transiently accelerated by interchanging to HPL culture medium. (**f**) Immunofluorescence analysis of the actin cytoskeleton (green) and the focal-adhesion associated protein vinculin (red). Representative images at day 4 (2 days interchange to HPL medium; nucleus counterstained with DAPI in blue). (**g**) The number of focal adhesions, (**h**) mean area of focal adhesions per cell, and (**i**) mean aspect ratio of the focal adhesions were quantified at day 4. FCS-MSCs have more elongated focal adhesions than HPL-MSCs, and this is reversible by interchanging culture media for two days (**p < 0.01; ****p < 0.0001; ns = not significant).

Since there was no significant difference in DNAm profiles of HPL-MSCs and FCS-MSCs we anticipated that the effects of the supplements on growth and cellular morphology might be rather transient - while the cells are in the corresponding culture medium. To address this question, we compared MSCs in parallel that were (i) continuously in FCS, (ii) continuously in HPL, or (iii) initially cultured in FCS, then cultured for two days in HPL, and then transferred back to FCS again (n = 4; Fig. 4a). Expression analysis of genes with the most pronounced differences in FCS-MSCs *versus* HPL-MSCs revealed that even short exposure to the opposite culture medium affects differential expression (Supplementary Figure S6). Furthermore, analysis of cell aspect ratios confirmed that FCS-MSCs were less elongated than HPL-MSCs (Fig. 4b). Already after two days interchanging to HPL culture medium the cells displayed a significantly more elongated phenotype (n > 250 cells/group; P-value < 0.0001; Fig. 4c). This effect on morphology could be changed back when the cells were subsequently again cultured in FCS (Fig. 4d). Furthermore, the proliferation of FCS-MSCs accelerated upon transfer into HPL medium, and again decelerated when transferred back to FCS medium (Fig. 4e).

Focal adhesions govern cellular morphology and mediate interaction of cells with extracellular matrix – and particularly such genes were differentially expressed in HPL-MSCs and FCS-MSCs, as mentioned above. In fact, immunofluorescent analysis of the focal adhesion protein vinculin and of the actin skeleton demonstrated that FCS-MSCs have more stress fibers spanning through the cytoplasm (Fig. 4f). Furthermore, FCS-MSCs had a significantly higher number of focal adhesions (Fig. 4g), that covered a larger area (Fig. 4h), and were more elongated (Fig. 4i) as compared to HPL-MSCs. These results indicate that MSCs in FCS have more intense interaction with the tissue culture plastic and exert higher tension forces. Notably, when cells that were initially cultured in FCS were transferred to HPL for two days, they adopted similar focal adhesions as those that were continuously cultured in HPL. These results demonstrate that the differences in proliferation rate, cellular morphology, and cell-biomaterial interaction are reversible by interchanging culture conditions.

## Discussion

Precise definition of cell preparations is essential for reproducible results – and this applies particularly to MSCs intended for therapeutic application. There have been attempts to better define MSCs based on proteomics and gene expression profiles^17, 18^. In this respect, it has to be taken into account that culture conditions impact on growth, morphology, transcriptome and proteome of MSCs^19–21^. Many studies have demonstrated that proliferation of MSCs is higher in HPL than in FCS^9, 22^ and that they reveal a more spindle-shaped fibroblast-like morphology^23, 24^. MSCs are heterogeneous^8, 25–27^ and if the different subsets have different response to the stimulatory effect of HPL then this would notoriously entail a shift in the cellular composition.

DNA-methylation patterns are ideally suited to define cell subpopulations since they are highly reproducible and can be quantified at a single nucleotide resolution. We have recently described a simple Epi-MSC-Score to discern fibroblasts and MSCs based on DNAm at two CpGs^12^. In that study, we have also compared publically available DNAm profiles of studies with either FCS-MSCs or HPL-MSCs and identified several differentially methylated CpGs^12^. The discrepancy can be attributed to the fact, that none of the previous studies directly compared HPL-MSCs and FCS-MSCs, and hence many other parameters may have confounding effects on this inter-study comparison. Our present study indicates that HPL-MSCs and FCS-MSCs are epigenetically identical. For our study design, it was important to use only one batch of FCS because otherwise additional variation between batches might have disguised the difference to HPL. It is conceivable that different batches of FCS or HPL might select for different subsets – but with regard to the similarity of cells isolated with very different types of supplements this appears less likely. In analogy, there may also be differences between different HPL preparations^6^. The observed differences in cellular behavior were reversible by interchange of culture conditions. This has also been observed by Griffiths *et al*. in MSCs of later passage^24^, and the authors suggested that this change in morphology might reflect cellular rejuvenation. However, at least with regard to senescence-associated DNAm and gene expression patterns there is no evidence that HPL does rejuvenate MSCs. The differences may rather be due to protein adsorptions to the tissue culture plastic. In this study, we have compared MSCs at passage two, because at this point clinically relevant cell numbers can already achieved and further culture expansion would have been notoriously associated with additional changes in morphology, function, and senescence-associated DNAm.

Our results indicate that culture media with HPL and FCS do not select for different cell populations. Albeit effects evoked by the supplements appear to be reversible, they may still be functionally and clinically relevant – particularly during *in vitro* culture and in the early phase of transplantation, when the cells need to adapt to a new environment. While the debate about ideal culture conditions will continue, at least the DNAm profiles demonstrate that the cellular composition is not affected by either HPL or FCS.

## Methods

### Cultivation of MSCs

Mesenchymal stromal cells were isolated from the bone marrow of six donors (ranging from 54 to 82 years) after orthopedic hip replacement surgery. All samples were taken after informed and written consent of all participants. The study and the experimental design were approved of the local ethics committees (donor 1–4: RWTH Aachen; EK300/13; donor 5–6: University of Würzburg OBELICS 100/14) and all methods were performed in accordance with the relevant guidelines and regulations. Cells were isolated as described before^15^. In brief, cells were flushed from the bone and cultured in parallel in basal medium with either 10% FCS or 10% HPL in a seeding density of 10,000 cells/cm^2^. Basal medium consisted of Dulbecco’s Modified Eagle Medium (DMEM, 1 g/L glucose; PAA, Pasching, Austria) with 1% penicillin/streptomycin (PAA) and 1% L-glutamine. Based on an initial screen of 11 FCS batches (Supplementary Figure S1) all experiments were performed with FCS from Bio&Sell (Lot number: 211507.5 A; Feucht, Germany) to exclude variation. HPL was generated as described previously^6, 28^. In brief, platelet units were generated by apheresis using the Trima Accel Collection System (CaridianBCT, Garching, Germany), aliquots of 45 mL were twice frozen at −80 °C and re-thawed at 37 °C, and then centrifuged at 2,600 × *g* for 30 minutes. The supernatant was filtered through 0.2 µm GD/X PVDF filters (Whatman, Dassel, Germany) and stored at −80 °C until use. To reduce variation we used HPL-pools consisting of at least 5 lysates. Coagulation was prevented by 0.61 IU unfractionated heparin (UFH; Ratiopharm, Ulm, Germany).

### Flow Cytometry Analysis

Immunophenotypic surface marker analysis was performed on a FACS Canto II (BD, Heidelberg, Germany) upon staining with CD14 allophycocyanin (APC, clone M5E2; BD), CD29 phycoerythrin (PE, clone MAR4; BD), CD31 PE (clone WM59; BD), CD34 APC (clone 8G12; BD), CD45 APC (clone HI30; BD), CD73 PE (clone AD2; BD), CD90 APC (clone 5E10; BD), CD105 fluorescein isothiocyanate (FITC, clone MEM-226; ImmunoTools, Friesoythe, Germany).

### Analysis of Proliferation, *in vitro* differentiation, viability and SA-βgal

Cells were seeded in defined numbers, after expansion harvested by trypsinization and counted in a Neubauer cell chamber^22^. Adipogenic and osteogenic differentiation capacity of MSCs was validated at passage 2 for all donors and for all culture conditions, as previously described^27^. Live/dead staining was performed with fluorescein diacetate (FDA) and propidium iodide (PI). For staining of the senescent cells the Abcam senescence detection kit was used according to manufacturer’s instructions (Abcam, Cambridge, UK).

### DNA-methylation analysis

Genomic DNA of MSCs was harvested with the NucleoSpin Tissue kit (Macherey-Nagel, Düren, Germany), bisulfite converted with the EZ DNA Methylation^TM^ Kit (Zymo Research, Irvine (CA), USA), and then analyzed on Infinium HumanMethylation450 BeadChip (Illumina, San Diego, CA, USA). Hybridization and initial data processing with the GenomeStudio (v2011.1) Methylation Module (v1.9.0) was performed at Life&Brain (Bonn, Germany). Raw data has been deposited at NCBIs Gene Expression Omnibus (GEO, http://www.ncbi.nlm.nih.gov/geo/, accession number: GSE87797).

### RNA extraction, qRT-PCR analysis, and Gene Expression Profiles

RNA was isolated from the samples using the NucleoSpin RNA extraction kit (Macherey-Nagel, Düren, Germany) and analyzed with a NanoDrop ND-1000 spectrophotometer (Thermo Scientific, Waltham, MA, USA) and an Agilent 2100 Bioanalyzer (Agilent Technologies, Santa Clara, CA, USA). Semi-quantitative qRT-PCR was carried out with the *Power SYBR* green master mix (Applied Biosystems, Foster City, CA, USA) and normalized to *Beta Actin* (Supplementary Table S3). For gene expression profiling 1 µg of genomic RNA was hybridized to GeneChip® Human Transcriptome Array 2.0 (Affymetrix, Santa Clara, USA). Raw data were normalized with Robust Multichip Average (RMA) using the oligo R package. These data have also been deposited at GEO (GSE87796). For principle component analysis (PCA) and nonsupervised hierarchical clustering (Euclidean distance) we used the entire gene set without X and Y chromosomes.

### Cell aspect ratio and fluorescence microscopy

The cellular aspect ratio (AR; total cellular length:width) of MSCs was determined in phase-contrast images (EVOS, Thermo Fisher Scientific) using the ImageJ software. Vinculin was stained with the antibody clone hVin1 (Sigma Aldrich, Hamburg, Germany) and actin was stained with Alexa Fluor488 phalloidin (Life Technologies, Darmstadt, Germany) and analyzed as described before^29^.

### Statistical Analysis

Statistical tests were performed using R bioconductor package or GraphPad Prism 6 statistical software. Data are represented as arithmetic mean ± standard deviation. Data were tested for normality and equal variance before analysis. Significance of DNAm and gene expression changes was estimated using the R limma package with an adjusted paired p-value < 0.05.

## Electronic supplementary material

Supplementary information
