# Supplementary material for: Human Platelet Lysate versus Fetal Calf Serum: These Supplements Do Not Select for Different Mesenchymal Stromal Cells
Source: Sci Rep. 2017 Jul 11;7:5132. doi: 10.1038/s41598-017-05207-1 (PMC5506010; doi:10.1038/s41598-017-05207-1)
Supplement: Supplementary file 1 — Supplementary information [file 41598_2017_5207_MOESM1_ESM.pdf]

Supplementary Material

Human Platelet Lysate versus Fetal Calf Serum: These Supplements Do Not Select for Different Mesenchymal Stromal Cells

Eduardo Fernandez-Rebollo, Birgit Mentrup, Regina Ebert, Julia Franzen, Giulio Abagnale, Torsten Sieben, Alina Ostrowska, Per Hoffmann, Pierre-François Roux, Björn Rath, Michele Goodhardt, Jean-Marc Lemaitre, Oliver Bischof, Jakob Franz, Wolfgang Wagner

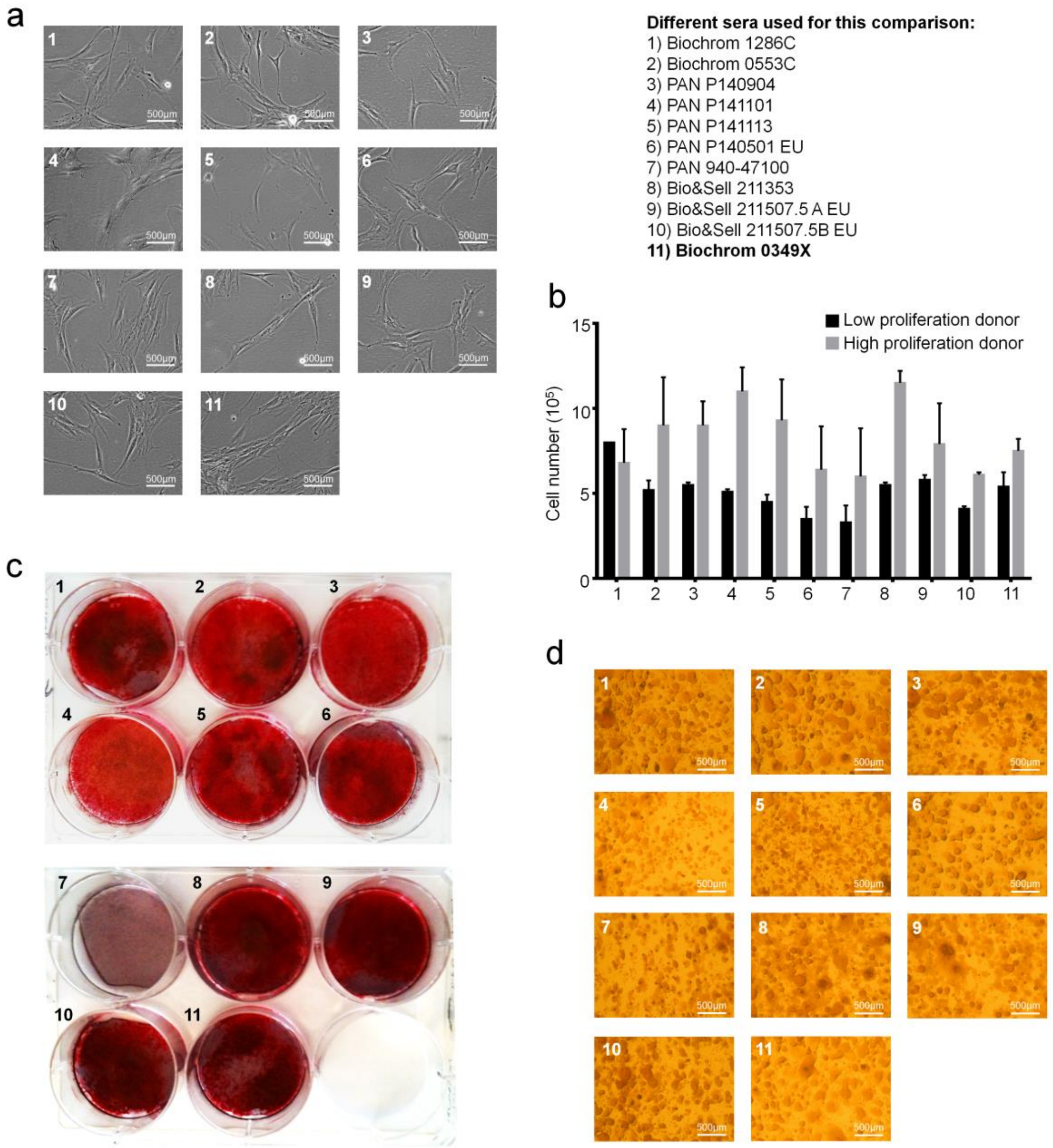

Supplementary Figure S1: Comparison of different FCS batches on growth and differentiation of MSCs. Mesenchymal stromal cells of one donor with a low and one donor with a high proliferation rate were cultured in parallel for one passage in eleven different FCS preparations that were purchased by three different companies.

(a) The growth pattern differed in FCS batches, whereas the cellular morphology of MSCs was rather similar.

(b) We have then seeded 1.8 x 10^5 and 2.7 x 10^5 cells in parallel in T25 flasks and manually counted them after three days (Error bars depict variation of two technical replicas).

(c) Osteogenic differentiation was induced for four weeks and then analyzed by Alizarin Red S staining. The photos exemplarily depict the variation with different FCS batches.

(d) Adipogenic differentiation was induced for two weeks and then analyzed by Oil Red O staining. The images exemplarily depict variation with different FCS batches.

Overall, the different FCS preparations varied in their support for growth and differentiation. Based on our results (and the costs) we decided to perform the subsequent experiments with FCS#11 (Biochrom 0349X; highlighted in bold).

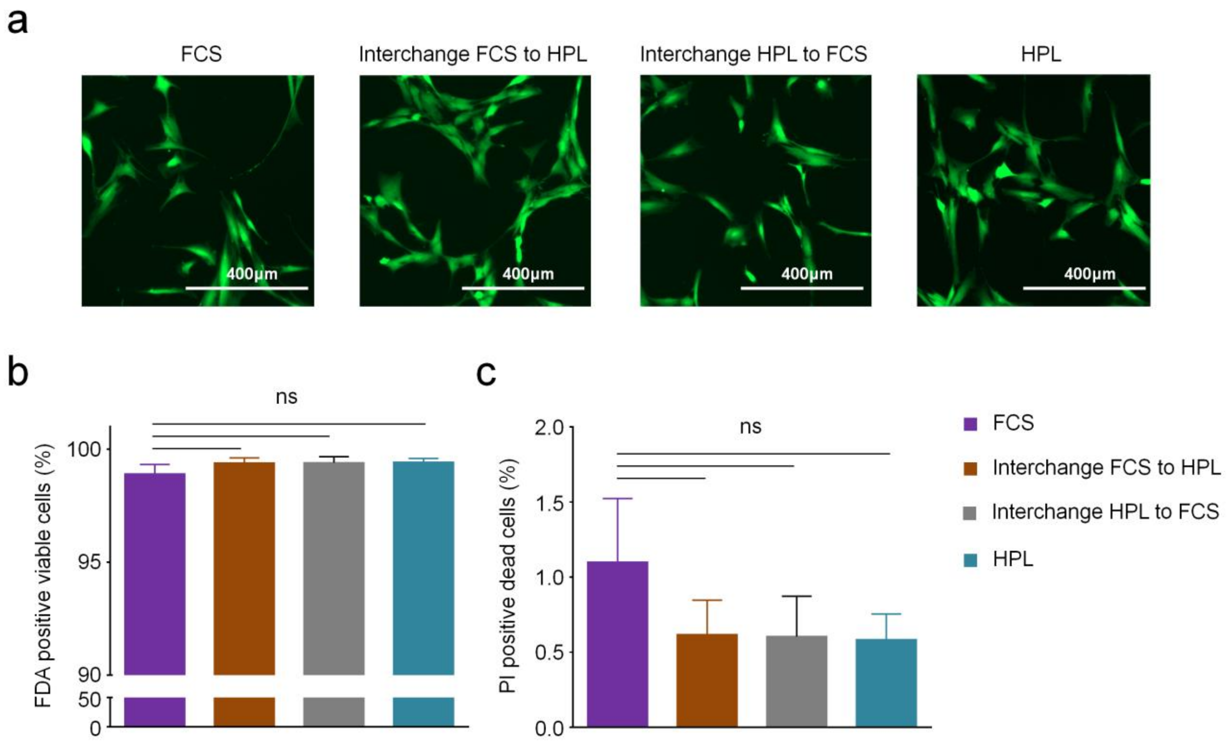

Supplementary Figure S2: Viability analysis of MSCs.
(a) Live/dead staining of MSCs with FDA (living cells, green) and PI (dead cells, red) for the four medium supplementation conditions.
(b) The quantification of the FDA/PI staining did not reveal any statistical differences (n = 4).

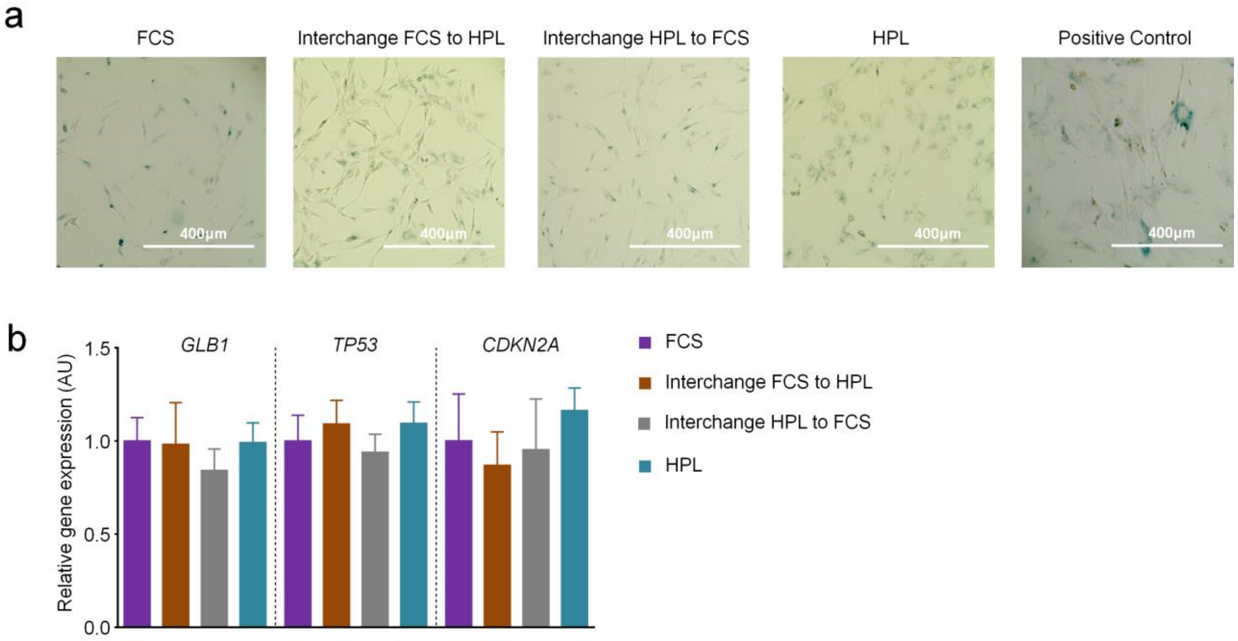

Supplementary Figure S3: Analysis of senescence by staining for SA-βgal or expression of marker genes.
(a) Senescence associated β-galactosidase (SA-βgal) staining at passage three did not reveal differences between the MSCs cultured with either FCS or HPL (positive control are senescent cells at passage 13).
(b) qRT-PCR analysis of the coding genes for β-galactosidase (*GLB1*)[1,2], P53 (*TP53*) and P16 (*CDKN2A*) showed no differences between FCS-MSCs, HPL-MSCs at passage three. For comparison we have also included MSC preparations with interchange of culture conditions for one passage (n = 4).

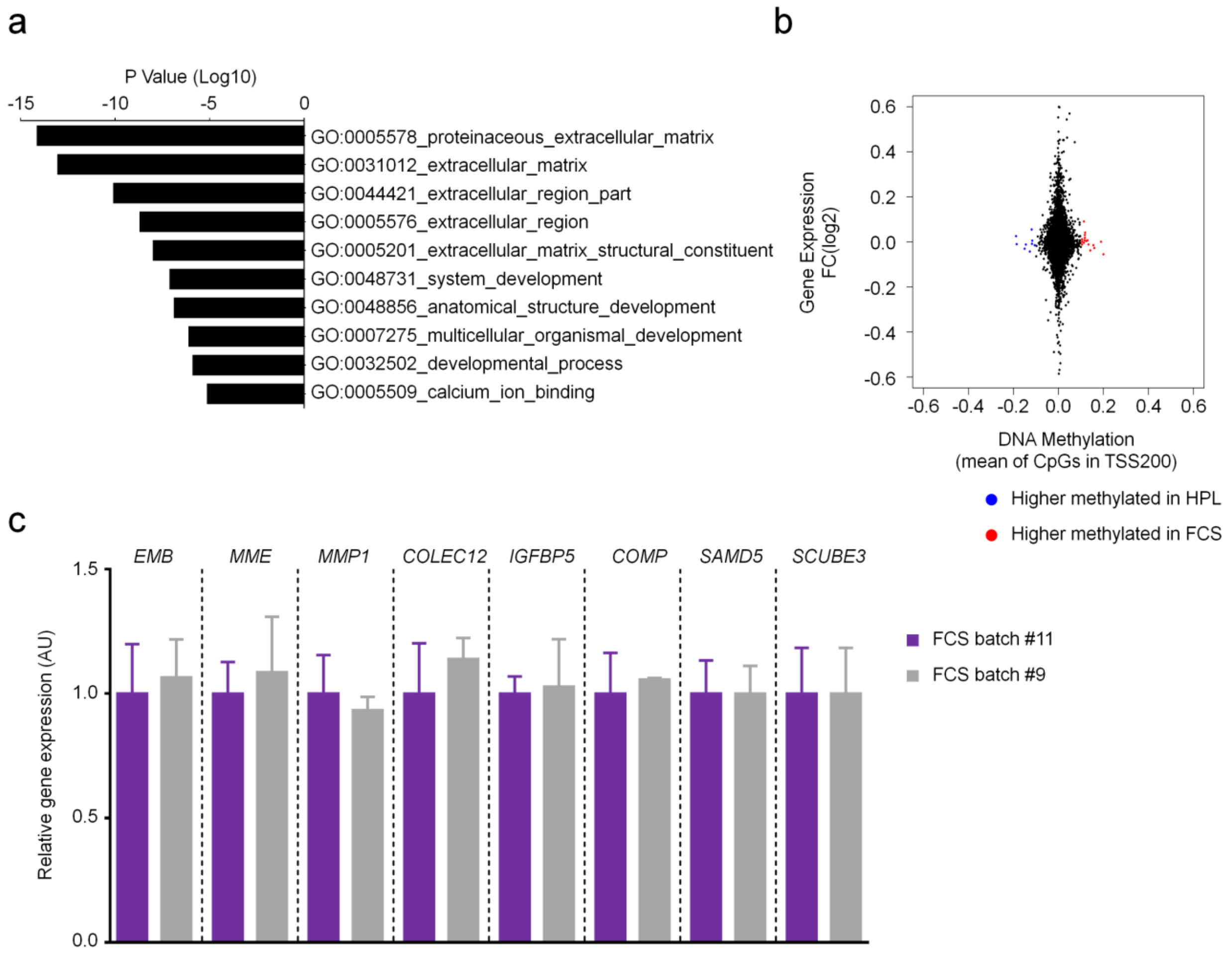

Supplementary Figure S4: Differentially expressed genes in FCS-MSCs *versus* HPL-MSCs.

(a) Gene ontology analysis of the differentially expressed genes revealed enrichment for extracellular matrix categories (analysis was performed with GoMiner tool).

(b) Scatter plot analysis of differences in DNAm as compared to gene expression changes of corresponding genes. For this comparison we utilized the mean DNAm levels of CpGs on the 450k BeadChip that correspond to each promoter region (200 base pairs upstream of transcription start site; TSS200). The difference of this mean TSS200 DNAm in FCS-MSCs *versus* HPL-MSCs was then compared to differential gene expression of the corresponding genes. Genes that comprised CpGs that are higher methylated in FCS and HPL (only 10% cutoff as depicted in Figure 2c) are indicated in red and blue, respectively. Overall, there was no clear association of mean DNAm in the promoter and corresponding gene expression – which may also be attributed to the very moderate (and non-significant) differences in DNAm.

(c) qRT-PCR analysis of the main deregulated genes in two different FCS batches did not reveal any statistical differences (n = 6 for batch#11 and n = 3 for batch#9).

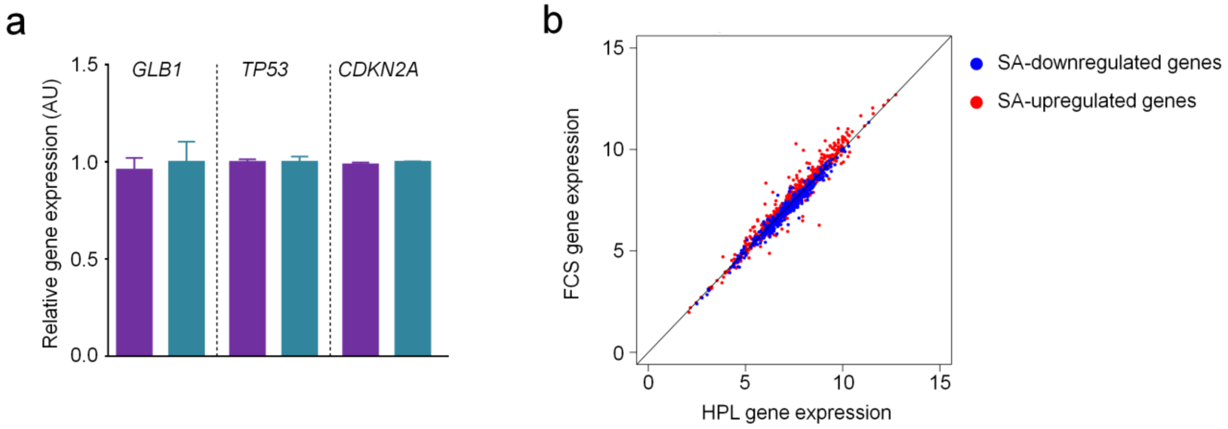

Supplementary Figure S5: Analysis of senescence-associated genes in microarray data.
(a) Gene expression from the HTA 2.0 array of the coding genes for β-galactosidase (*GLB1*)[1,2], P53 (*TP53*) and P16 (*CDKN2A*) showed no differences between HPL or FCS supplementation (n = 6).
(b) Various senescence-associated (SA) genes were previously described to be up- or downregulated in MSCs during replicative senescence (981 upregulated and 631 downregulated)[3]. Scatterplot analysis of these genes in our datasets did not reveal differences between FCS-MSCs and HPL-MSCs (n = 6).

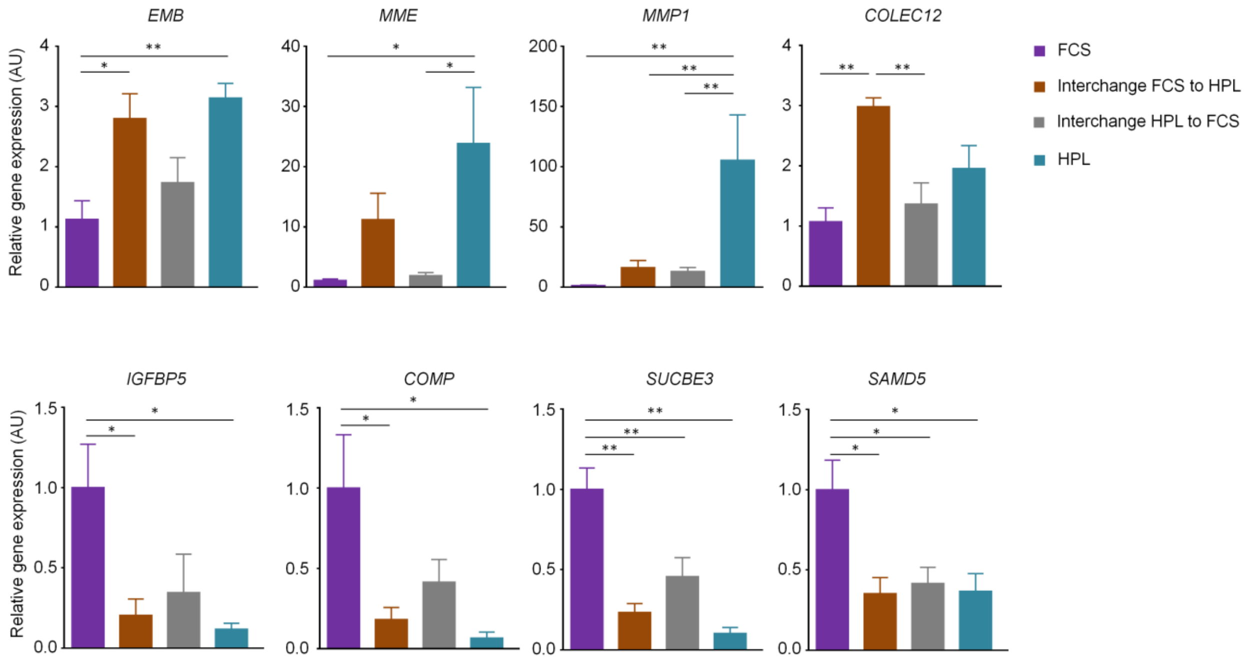

Supplementary Figure S6: Short interchange of culture conditions impacts on gene expression.
Gene expression of eight genes that were either higher expressed in microarray data of HPL-MSCs (*EMB*, *MME*, *MMP1*, and *COLEC12*) or FCS-MSCs (*IGFBP5*, *COMP*, *SUCBE3*, and *SMAD5*) were exemplarily analyzed by qRT-PCR. Differential gene expression was validated for all of these genes in FCS-MSCs versus HPL-MSCs. Notably, even interchange of culture conditions for two days impacted at least in tendency on differential gene expression.

Supplementary Table S1: Publically available datasets of MSCs isolated in FCS or HPL.

GSE number	Reference	Samples with FCS	Samples with HPL	Age mean (min/max)	Gender (m/f)	Tissue of origin
**GSE74609**	4	4	0	43 (43/43)	4/0	BM (from Lonza)
**GSE41933**	5	0	6	NA (18/39)	6/0	AT, UC, BM
**GSE55888**	6	0	8	NA	NA	AT
**GSE37066**	7	0	5	NA (31/73)	NA	BM
**GSE52114**	8	34	0	37 (2/92)	7/27	BM
**GSE55571**	9	1	0	NA	1/0	NA

BM = Bone marrow; AT = adipose tissue; UC = umbilical cord; NA = not available. Reference numbers correspond to the additional supplementary references.

Supplementary Table S2: Differentially expressed genes in FCS-MSCs versus HPL-MSCs.

Affymetrix ID	logFC	adj.P.Val	Gene ID
**TC02002758.hg.1**	-4.39	7.6E-05	*IGFBP5*
**TC15000842.hg.1**	-4.19	4.6E-05	*ACAN*
**TC15000843.hg.1**	-3.76	6.7E-05	*ACAN*
**TC19001293.hg.1**	-2.85	0.0071	*COMP*
**TC06003119.hg.1**	-2.74	0.0198	*SAMD5*
**TC06000511.hg.1**	-2.68	0.0021	*SCUBE3*
**TC15002348.hg.1**	-2.58	0.0002	*ACAN*
**TC06000126.hg.1**	-2.57	0.0020	*ID4*
**TC09000388.hg.1**	-2.53	0.0003	*NTRK2*
**TC07002404.hg.1**	-2.51	0.0017	*ELN*
**TC01001686.hg.1**	-2.46	0.0065	*PRELP*
**TC01003722.hg.1**	-2.39	0.0050	*FMOD*
**TC20000621.hg.1**	-2.27	0.0001	*JAG1*
**TC20001421.hg.1**	-2.24	0.0012	*JAG1*
**TC06001189.hg.1**	-2.23	0.0020	*SMOC2*
**TC15002695.hg.1**	-2.16	0.0108	
**TC15001823.hg.1**	-2.12	0.0022	
**TC12002778.hg.1**	-2.07	0.0230	*MGP*
**TC06002124.hg.1**	-2.07	0.0082	*SLC2A12*
**TC02001750.hg.1**	-2.02	0.0022	*CYP1B1*
**TC10001522.hg.1**	-2.00	0.0152	*PPP1R3C*
**TC20000927.hg.1**	-1.97	0.0148	*PTGIS*
**TC11002859.hg.1**	-1.94	0.0174	*C11orf87*
**TC12001181.hg.1**	-1.93	0.0190	*MFAP5*
**TC07000449.hg.1**	-1.92	0.0075	*ELN*
**TC10002843.hg.1**	-1.87	0.0104	*GFRA1*
**TC12000060.hg.1**	-1.85	0.0018	*CCND2*
**TC12001801.hg.1**	-1.82	0.0239	*EPYC*
**TC02001749.hg.1**	-1.79	0.0078	*CYP1B1*
**TC12001276.hg.1**	-1.78	0.0110	*MGP*
**TC08002329.hg.1**	-1.77	0.0322	*LOC100507516*
**TC06000960.hg.1**	-1.76	0.0022	*HEY2*
**TC10002842.hg.1**	-1.66	0.0016	
**TC21000892.hg.1**	-1.66	0.0309	*ADAMTS5*
**TC04001504.hg.1**	-1.63	0.0071	*PDE5A*
**TC06003061.hg.1**	-1.63	0.0198	*EYA4*
**TC04000254.hg.1**	-1.62	0.0013	*LIMCH1*
**TC06001072.hg.1**	-1.62	0.0259	*SAMD5*
**TC08002397.hg.1**	-1.61	0.0065	*ZNF704*
**TC19001250.hg.1**	-1.60	4.6E-05	*NOTCH3*
**TC09002022.hg.1**	-1.61	0.0207	
**TC02003890.hg.1**	-1.59	0.0021	
**TC12000624.hg.1**	-1.58	0.0116	*LGR5*
**TC05002693.hg.1**	-1.55	0.0296	
**TC21000346.hg.1**	-1.53	0.0038	*ADAMTS5*
**TC08001214.hg.1**	-1.52	0.0423	*LOC100507516*
**TC10001133.hg.1**	-1.52	0.0035	*MKX*
**TC12001933.hg.1**	-1.50	0.0193	*CMKLR1*
**TC01002807.hg.1**	1.50	0.03378	*ELTD1*
**TC12003096.hg.1**	1.51	0.03708	*TBX3*
**TC01005156.hg.1**	1.52	0.02108	
**TC03001924.hg.1**	1.59	0.0414	*MME-AS1*
**TC10002885.hg.1**	1.64	0.0039	
**TC12001751.hg.1**	1.66	0.0075	*PHLDA1*
**TC06000846.hg.1**	1.70	0.0015	*AIM1*
**TC01001053.hg.1**	1.74	0.0075	*EMBP1*
**TC11002235.hg.1**	1.80	0.0256	*MMP3*
**TC11002175.hg.1**	1.82	0.0135	*CTSC*
**TC10001389.hg.1**	1.92	0.03098	
**TC05001331.hg.1**	1.95	0.0064	*EMB*
**TC05002946.hg.1**	2.00	0.0106	*ADAMTS12*
**TC05002960.hg.1**	2.01	0.03025	*GDNF*
**TC01004676.hg.1**	2.18	0.0014	*EMBP1*
**TC11003312.hg.1**	2.45	0.03386	*MMP1*
**TC06000985.hg.1**	2.45	0.0181	*ENPP1*
**TC05002988.hg.1**	2.49	0.0050	*EMB*
**TC03000846.hg.1**	2.53	0.0422	*MME*
**TC11002234.hg.1**	2.66	0.0345	*MMP1*
**TC18000273.hg.1**	2.99	0.0211	*COLEC12*

Supplementary Table S3: Table for qRT-PCR primers.

Name	5' - 3'	Size (bp)
qhFw-EMB	GAGTGTAAAGGTTCCTGTTGGT	126
qhRv-EMB	GCACGGCACCAGTAAGATT	
qhFw-MME	TGCAACCTACGATGATGGTATT	102
qhRv-MME	AAGTCTGTACAAGGCTCAGTG	
qhFw-MMP1	CACATCTGACCTACAGGATTGAA	129
qhRv-MMP1	CCTCAGAGACCTTGGTGAATG	
qhFw-COLEC12	TGAAAGACGACTTCGCAGAG	76
qhRv-COLEC12	TGTGTTCCTTCCTGAATACCAA	
qhFw-IGFBP5	GAGCAAGTCAAGATCGAGAGAG	114
qhRv-IGFBP5	GGAGATGCGGGTGTGTTT	
qhFw-COMP	GTCAACGAGTGCAACGC	149
qhRv-COMP	ACCTGCTTGTTGGCCTT	
qhFw-SAMD5	CCCAAACTGAAGCTGAAGATCA	98
qhRv-SAMD5	CCAGCCATTGGGACCTTG	
qhFw-SCUBE3	ACTGCAAAGACGTGGATGAG	95
qhRv-SCUBE3	ATAGCAGGTACACCGGTAATTG	
qhFw-GLB1	ATCTCAGGAAGCATTCACTACTC	100
qhRv-GLB1	CATACGTCTGGATGGCGTT	
qhFw-ACTB	GGCACCACACCTTCTACAAT	115
qhRv-ACTB	AACATGATCTGGGTCATCTTCTC	
qhFw-CDKN2A	GGTCGGGTAGAGGAGGT	95
qhRv-CDKN2A	ATCATCATGACCTGGATCGG	
qhFw-TP53	CAGACCTATGGAAACTACTTCCTG	117
qhRv-TP53	CTGGGTCTTCAGTGAACCATT	

Additional References for Supplementary Material

1. Lee, B. Y. *et al.* Senescence-associated β-galactosidase is lysosomal β-galactosidase. *Aging Cell* **5,** 187–195 (2006).
2. Debacq-Chainiaux, F. *et al.* Screening of senescence-associated genes with specific DNA array reveals the role of IGFBP-3 in premature senescence of human diploid fibroblasts. *Free Radic. Biol. Med.* **44,** 1817–1832 (2008).
3. Wagner, W. *et al.* Aging and replicative senescence have related effects on human stem and progenitor cells. *PLoS One* **4,** e5846 (2009).
4. van den Dungen, M. W., Murk, A. J., Kok, D. E. & Steegenga, W. T. Comprehensive DNA Methylation and Gene Expression Profiling in Differentiating Human Adipocytes. *J. Cell. Biochem.* **9999,** 1–12 (2016).
5. Reinisch, A. *et al.* Epigenetic and in vivo comparison of diverse MSC sources reveals an endochondral signature for human hematopoietic niche formation. doi:10.1182/blood-2014-04
6. Schellenberg, A. *et al.* Matrix elasticity, replicative senescence and DNA methylation patterns of mesenchymal stem cells. *Biomaterials* **35,** 6351–8 (2014).
7. Koch, C. M. *et al.* Pluripotent stem cells escape from senescence-associated DNA methylation changes. *Genome Res.* **23,** 248–59 (2013).
8. Fernández, A. F. *et al.* H3K4me1 marks DNA regions hypomethylated during aging in human stem and differentiated cells. *Genome Res.* **25,** 27–40 (2015).
9. Miyata, K. *et al.* DNA methylation analysis of human myoblasts during in vitro myogenic differentiation: de novo methylation of promoters of muscle-related genes and its involvement in transcriptional down-regulation. *Hum. Mol. Genet.* **24,** 410–23 (2015).
